# Prevalence of substandard, falsified, unlicensed and unregistered medicine and its associated factors in Africa: a systematic review

**DOI:** 10.1080/20523211.2024.2375267

**Published:** 2024-07-15

**Authors:** Biset Asrade Mekonnen, Muluabay Getie Yizengaw, Minichil Chanie Worku

**Affiliations:** a Department of Pharmacy, College of Medicine and Health Sciences, Bahir Dar University, Bahir Dar, Ethiopia; b Biochemistry, College of Medicine and Health Sciences, Bahir Dar University, Bahir Dar, Ethiopia; c Department of Pharmaceutical Chemistry, School of Pharmacy, College of Medicine and Health Sciences, University of Gondar, Gondar, Ethiopia

**Keywords:** Counterfeit, falsified, fake, substandard, unlicensed, unregistered, drugs, medicine, Africa

## Abstract

**Background:**

Substandard, falsified, unlicensed, and unregistered medicines pose significant risks to public health in developed and developing countries. This systematic review provides an overview of the prevalence of substandard, falsified, unlicensed, and unregistered medicine and its associated factors in Africa.

**Methods:**

Articles published from April 2014 to March 2024 were searched in Google Scholar, Science Direct, PubMed, MEDLINE, and Embase. The search strategy focused on open-access articles published in peer-reviewed scientific journals and studies exclusively conducted in African countries. The quality of the studies was assessed according to the Medicine Quality Assessment Reporting Guidelines (MEDQUARG). This systematic review was reported according to the Preferred Reporting Items for Systematic Reviews and Meta-Analysis (PRISMA).

**Results:**

Of the 27 studies, 26 had good methodological quality after a quality assessment. Of the 7508 medicine samples, 1639 failed at least one quality test and were confirmed to be substandard/falsified medicines. The overall estimated prevalence of substandard/falsified medicines in Africa was 22.6% (1718/7592). The average prevalence of unregistered medicines was 34.6% (108/312). Antibiotics, antimalarial, and antihypertensive medicines accounted for 44.6% (712/1596), 15.6% (530/3530), 16.3% (249/1530), and 16.3% (249/1530), respectively. Approximately 60.7% (91/150) were antihelmintic and antiprotozoal medicines. Poor market regulatory permission, Free trade zones, poor registration, high demand, and poor importation standards contribute to the prevalence of these problems.

**Conclusion/Recommendations:**

Substandard, falsified, and unregistered medicines are highly prevalent in Africa, and attention has not been paid to the problem. Antibiotics, antimalarial, anthelmintic, and antiprotozoal are the most commonly reported substandard, falsified, and unregistered medicines. A consistent supply of high-quality products, enhancement of registration, market regulatory permission, and importation standards are essential to counter the problems in Africa. Preventing these problems is the primary duty of every responsible nation to save lives.

## Introduction

Medicines can save lives and prevent acute and chronic diseases only if they are safe, efficacious, of good quality, and used rationally(Sweileh, 2021; WHO, 2017a). However, substandard, falsified, unlicensed, and unregistered medicines have been reported in all therapeutic categories, including medicines, vaccines, and in vitro diagnostics, which are harmful to the health of patients and the general population (Johnston & Holt, 2014; Kelesidis & Falagas, 2015; Meeking, 2013; Sweileh, 2021; USTR, 2011; WHO, 2017a).

Medicine is falsified when its identity and/or source are falsely represented. This applies to products, containers, and other packaging or labelling information. Falsifying can apply to both branded and generic products, and falsified medicines include products with correct or incorrect components, without active ingredients, with incorrect amounts of active ingredients, or with fake packaging (Hauk et al., 2021; UNODC, 2019; WHO, 2017a).

Other categories of medicines of international concern, such as substandard medicines or out-of-specification medicines are medicines that do not meet the quality standards specified for them and unregistered or unlicensed medicines also medicines that have not undergone evaluation and/or approval following the national or regional regulations and legislation for the market in which they are marketed or distributed or used, subject to permitted conditions under national or regional regulation and legislation(Hauk et al., 2021; UNODC, 2011, 2019; WHO, 2017b).

The WHO estimated that half of the world’s population did not have regular access to essential medicines, with this number reducing to 33% in Sub-Saharan Africa (WHO, 2017b). In particular, this is an important issue in Africa. There are many constraints to the regular supply of essential medicines, including geographic location, logistics, ensuring the integrity of the supply chain, and financing the supply of essential medicines, which play a role in the distribution of substandard, falsified, unlicensed, and unregistered medicines in Africa (Akinyandenu, 2013; Morris & Stevens, 2006; WHO, 2017b).

Substandard and falsified medicines are complex but critical global health issues. The World Health Organization (WHO) estimates that 10% of medicines worldwide are substandard or falsified and fail quality testing in low- and middle-income countries (WHO, 2017b). Falsified medicines could comprise approximately 50% of the drug market worldwide; many of these products are sourced from developing countries (Glass, 2014; WHO, 2017b). According to the Organization for Economic Co-operation and Development (OECD) report, the annual cost of the global trade of falsified medicines is approximately 4.4 billion USD (OECD and EUIPO, 2019). The global trade of substandard and falsified medicines is also increasing daily in low- and middle-income countries because consumer demand for medicines is also increasing (Anon, 2020; Mia & Mallick, 2021; OECD and EUIPO, 2019). This indicates that medicines do not meet quality specifications and are deliberately substandard or falsified and circulated in the market (Bekoe et al., 2020; Ozawa et al., 2018; Peyraud et al., 2017; WHO, 2017b).

Substandard, falsified, unlicensed, and unregistered medicines are by their very nature, difficult to detect. They are often designed to appear identical to genuine medicine; however, they often have many public health and socioeconomic impacts in both developing and developed countries (EUIPO, 2020; OECD, 2020; Peyraud et al., 2017; WHO, 2017c). They are a major cause of morbidity and mortality due to treatment failure, adverse drug reactions, drug resistance development, economic strain, and loss of confidence in medicine, healthcare providers, and various health services, as well as in those who manufacture, distribute, and dispense or sell products (Cui, 2012; Wertheimer, 2012; WHO, 2017d).

Studies in 2018 found 48 reported incidents, 56.3% of which occurred in developing countries and 43.7% in developed countries, affecting approximately 7,200 consumers and resulting in 3,604 deaths (Rahman et al., 2018). The use of substandard, falsified, and unregistered anti-malarial medicines has many public health implications and is responsible for 12,300 deaths per year in Nigeria. Due to the development of anti-malarial resistance, treatment efficacy was decreased, and the cost of malaria treatment increased by 11%, which has a total economic impact of 11% and leads to 11% of total productivity loss (Beargie et al., 2019).

Understanding the prevalence of substandard, falsified, unlicensed, and unregistered medicine in Africa is essential for the development and implementation of initiatives. To make informed decisions and improve a country’s supply chain and drug post-market surveillance systems, concerned bodies must be aware of the extent to which substandard, falsified, unlicensed and unregistered medicines are introduced into the market. Hence, the findings of this review may help to identify factors contributing to the problem’s distribution throughout Africa as well as informing policymakers, the World Health Organization, the Minister of Health, regulatory bodies, researchers, and healthcare professionals about the magnitude of the problem and how to prevent and reduce its distribution in Africa.

## Methods

### Search strategy

**Figure 1.**
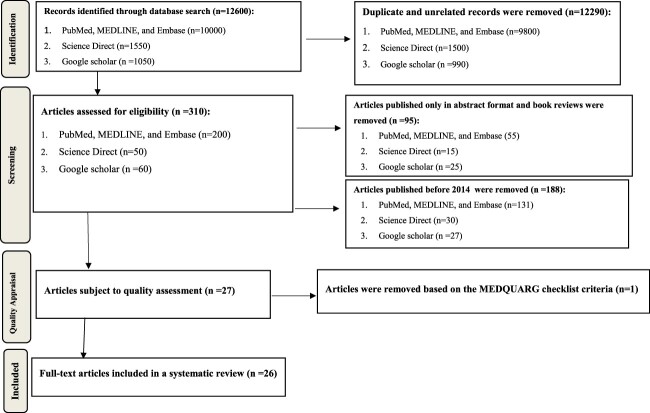
Systematic review and search process ﬂow diagram based on the PRISMA guidelines.

The Preferred Reporting Items for Systematic Reviews and Meta-Analysis (PRISMA) was used to search for articles) (Moher et al., 2009). The search was limited to articles published from April 2014 to April 2024 using keywords. The chosen time frame was sufficiently wide to capture the breadth of articles and was also sufficiently narrow to exclude articles that might no longer be relevant. The entire search was conducted from March 1 to 31, 2024, using stepwise procedures. The initial advanced search was performed in Google Scholar, Science Direct, PubMed, MEDLINE, and Embase. Articles identified by search and considered meeting the inclusion criteria and quality appraisal were obtained for data analysis (Figure 1).

Keywords: (substandard OR spurious OR falsified OR fake OR counterfeit OR unlicensed OR unregistered) AND (drug OR medicine OR pharmaceutical product OR medical product) AND (Africa OR African countries).

### Eligibility criteria

The review included open-access articles published in peer-reviewed scientific journals within the last ten years that were solely conducted in African countries, whereas book reviews, studies published only in abstract format or without full-text availability, and unrelated articles were excluded. The full search strategy and result flow diagram are shown in Figure 1.

### Quality evaluation assessment

The selected articles were subjected to further quality assessment using the Medicine Quality Assessment Reporting Guidelines (MEDQUARG) checklist. The MEDQUARG checklist of items to be addressed in reports of surveys on medicine quality was adopted to provide 12 criteria against which to assess the quality of each included study methodology (Newton et al., 2009).

This study was conducted to minimise bias associated with the methodology used for data collection, and the quality of the studies was evaluated. The methodological strength of the studies included in this review can be directly compared with those of other reviews, and a study must score between 6 and 12 according to the MEDQUARG checklist criteria to be considered methodologically sound and included in this systematic review. The following points were covered by the inclusion criteria and were essential to meet the intended scope of the study (Table 1) (Almuzaini et al., 2013; Mcmanus & Naughton, 2020; Newton et al., 2009).

**Table 1.** Medicine Quality Assessment Reporting Guidelines (MEDQUARG) checklist.

No	MEDQUARG Checklist
1.	Definition of substandard/falsified medicines
2.	Sample design and sample size calculation
3.	The chemical analysis is clearly described. Any paper meeting three of the nine additional criteria should be considered to have sufficient methodological rigour for inclusion in further analysis. These nine criteria are as follows:
4.	The timing and location of the study were clearly stated.
5.	The type of outlets sampled
6.	Type and number of dosage units purchased per outlet
7.	Random sampling was used
8.	Information about who collected the samples
9.	Packaging assessment performed
10.	The statistical analysis is described
11.	Details of method validation
12.	Chemical analysis was performed in subjects blinded to packaging

### Data extraction

The list of data to be extracted should be agreed upon a priori by consensus during the design stage of the study following the Medicine Quality Assessment Reporting Guidelines (MEDQUARG) and a recently published checklist of criteria for designing and reporting medicine quality studies (Almuzaini et al., 2013; Khras, 2016; Lalani et al., 2017; Moher et al., 2009; Mwamba et al., 2016; Newton et al., 2009; Peter et al., 2016). The inclusion and exclusion criteria were used to screen and evaluate each article. The full articles were then retrieved, and using a predesigned data extraction form, the following information was independently extracted from each study: study location, the year it was published, type of medicines sampled, sample size, dosage forms, sampling methods, analytical techniques employed, origin of the drugs, percentage and issues related to substandard, falsified, unlicensed, and unregistered medicines, MEDQUARG score, and all pertinent factors were taken into account. Then, articles were grouped according to whether they were quantitative, qualitative, or both, and a review-specific data extraction form was designed. Important and relevant findings were extracted from these 26 studies.

### Data analysis

After data extraction and collection from the various data sources and assessment of their quality and validity, the relevant findings from each study were entered into Microsoft Excel and subjected to analysis using SPSS version 20. Subsequently, descriptive data were pooled and presented in a table within the systematic review. However, very conflicting and widely variable data should not be combined numerically.

## Results

A total of 12600 publications were identified through database searches. After removing 12,290 duplicates, 310 articles were considered eligible. A quality assessment was conducted on 27 studies identified between 2014 and 2024 based on the eligibility criteria. After a quality assessment using the MEDQUARG checklist, 26 studies were found to have good methodological quality and were subsequently included in the analysis. The study samples were collected from randomly chosen outlets within a predetermined area, and pharmacopeia standards were followed for analysis. Compared with 32/33 studies in the 2018 review (97%), 4/15 studies in the 2013 review (27%), and 5/15 studies in the 2024 review (33.3%), 19 studies (73.1%) of the 26 studies used random sampling, while the remaining studies were purposeful and convenient (Afrassa et al., 2024; Almuzaini et al., 2013; Mcmanus & Naughton, 2020).

Of the 7508 medicine samples, 1639 failed at least one quality test and were confirmed to be substandard/falsified medicines. Samples mainly failed because they did not contain the correct amount of the active pharmaceutical ingredient (API), may not contain API, failed dissolution and content uniformity testing, reduced bioavailability, microbial contamination, and other failures. In Africa, the overall estimated prevalence of substandard/falsified medicines was 22.6% (1718/7592). The average prevalence of unregistered medicines was 34.6% (108/312). The average prevalence of antibiotics, antimalarial, and antihypertensive medicines was 44.6% (712/1596), 15.6% (530/3530), and 16.3% (249/1530), respectively. Approximately 60.7% (91/150) were antihelmintic and antiprotozoal medicines (Tables 2–5).

**Table 2.** Studies on substandard, falsified, unlicensed, and unregistered antibiotics in Africa.

Country	Sample size (n)	Sampling Medicines	Sampling Methods	Analytical Techniques	Major Findings	References
Uganda	145	Ceftriaxone (n = 36), Cefixime (n = 22), Cefuroxime (13), Cephalexin (n = 34),and Erythromycin (n = 40)	Convenience	HPLC-UV	17.24% were substandard/falsified ✓ 48%-Cefixime ✓ 12%-Cefuroxime ✓ 40%-Erythromycin	Kitutu (2015)
Congo	1025	Amoxicillin, Ampicillin, Ceftriaxone, Ciprofloxacin, Doxycycline, Ofloxacin, Sulfamethoxazole, Tetracycline, and Trimethoprim	Random	HPLC	60.2% outside uniformity of content limit range ✓ 67.1% Amoxicillin above the limit ✓ 58.8% Ciproﬂoxacin above the limit ✓ 57.4% Ofloxacin above the limit ✓ 57.1% Ceftriaxone above the limit ✓ 51.6% Trimethoprim above the limit ✓ 34.7% Sulfamethoxazole above the limit	Patricia et al. (2019)
Kenya	106	Co- trimoxazole suspension	Random	HPLC	13.2% were substandard/falsified	Njeri et al. (2021)
Ethiopia	9	Norfloxacin tablet	Convenience	HPLC	22.22% were substandard/falsified	Solomon et al. (2019)
Kenya	53	Amoxicillin and amoxicillin/clavulanic acid	Purposive	HPLC-UV	37.7% were substandard/falsified	Koech et al. (2020)
Ethiopia	10	Doxycycline Hyclate	Convenience	HPLC-UV	30% were substandard/falsified	Abraham et al. (2021)
Rwanda	232	Amoxicillin, Co-trimoxazole, Cloxacillin, Erythromycin, Ciprofloxacin, Metronidazole, Phenoxymethylpenicillin, Ampicillin, Ceftriaxone, Cefotaxime, and Amoxicillin + Clavulanic acid	Random	HPC-UV	8.2% were substandard/falsified	Bizimana et al. (2022)
Nigeria	16	Ciprofloxacin and metronidazole injction	Random	Membrane filtration method and UV	75% had poor quality 18.8% were non sterile	Oli et al. (2020)

HPLC-High performance liquid chromatography; UV-Ultra-Violent detectors.

**Table 3.** Studies on substandard, falsified, unlicensed, and unregistered antimalarial medicines in Africa.

Country	Sample size (n)	Sampling Medicines	Sampling Methods	Analytical Techniques	Major Findings	References
Ghana and Togo	132	ACT formulation (n = 90) and Artemisinin-based monotherapy formulations (n = 42)	Convenience	HPLC	75% were substandard/falsified 46% were not registered 84.7% of unregistered medicines were SF 70.8% of registered medicines were SF 77.3% of domestic manufacturing sources were SF 77.5% of foreign manufacturing sources were SF	Osei-Safo et al. (2014)
Tanzania	1737	Artemisinin-based drugs	Random	HPLC, MS &UV	4.1% were substandard/falsified	Act Consortium. (2015)
Ghana	254	ALU & Artesunate	Random	HPLC, MS & UV	35.0% were substandard	Tivura et al. (2016)
Congo	52	Artemether (n = 37) and Artesunate (n = 15)	Random	HPLC	12% were substandard 13% were falsified	Tshilumba1 et al. (2016)
Equatorial Guinea	677	artemisinin-containing antimalarials (ACAs)	Convenience	HPLC-DAD	6.1% were substandard 16.1% were falsified.	Kaur et al. (2017)
Ethiopia	60	Chloroquine Phosphate and Quinine Sulfate	Random	HPLC	6.8% were substandard/falsified	Abuye et al. (2020)
Gabon	432	Artemether-lumefantrine, sulfadoxine-pyrimethamine	Random	HPLC-DAD	0.5% were substandard/falsified	Visser et al. (2015)
Malawi	112	artemether and lumefantrine, artesunate, dihydroartemisinin	Random	HPLC	88.4% poor quality 13.4% not registered	Chikowe et al. (2015)
Ethiopia	74	FDC artemether/ lumefantrine	Random	HPLC-DAD	4% were substandard/falsified	Belew et al. (2019)

SF-substandard/falsified, ALU –Artemeter-Lumafantrine and SP-Sulfadoxine/Pyrimethamine, DAD-diode array detector.

**Table 4.** Studies on substandard, falsified, unlicensed, and unregistered antibiotics, antimalarial, and other medicines in Africa.

Country	Sample size (n)	Sampling Medicines	Sampling Methods	Analytical Techniques	Major findings	References
Papua New Guinea	360	Primaquine, amodiaquine, quinine, sulphadoxine-pyrimethamine amoxicillin, and artemether	Random	HPLC	9.7% were substandard 0.6% contains more API	Hetzel et al. (2014)
Tanzania	242	Amoxicillin, co-trimoxazole, artemether lumefantrine, quinine, ergometrine, and paracetamol	Random	HPLC	7.4% were substandard/falsified	Kaale et al. (2016)
Kenya	60	Ibuprofen, Amoxicillin/clavulanic acid, Cetirizine, Prednisolone, Salbutamol, and zinc	Convenience	HPLC	17% were substandard/falsified	Wafula et al. (2016)
Malawi	56	Amoxicillin, ALU and SP	Random	HPLC-UV	10.7% were substandard 1.9% were falsified	Khuluza et al. (2017)
Ghana	68	Ibuprofen, Amoxicillin, Mebendazole, Albendazole ciprofoxacin, Griseofulvin, cotrimoxazole, fucloxacillin, artemether-lumefantrine, Metronidazole, Ferrous sulphate, multivitamin, Cefuroxime, folic acid, and paracetamol	Random	HPLC-UV	61.8% had poor quality 47.1% were not registered 59.4% of imported products failed 63.9% of locally manufactured products failed 1.5% had no batch number	Frimpong et al. (2018)

**Table 5.** Studies on substandard, falsified, unlicensed, and unregistered medicines other than antibiotics and antimalarial medicines in Africa.

Country	Sample size (n)	Sampling Medicines	Sampling Methods	Analytical Techniques	Major Findings	References
Ethiopia	106	Mebendazole, Albendazole, and Tinidazole	Random	HPLC	45.3% were substandard 29.2% were falsified	Suleman et al. (2014)
Congo	34	Albendazole and Metronidazole tablet /suspension	Random	HPLC	26% were substandard 78% of substandard is non-authorised drugs	Mwamba et al. (2016)
Benin, Burkina Faso, Congo-Brazzaville, DRC, Senegal, Togo, Niger, Côte D’Ivoire, Guinea, and Mauritania	1530	Furosemide, HCT, Captopril, Simvastatin, and Amlodipine	Random	HPLC	16.3% were substandard/falsified	Faso (2017)
Ethiopia	10	Albendazole, Mebendazole, and Praziquantel	Random	HPLC-UV	33.33% had poor quality	Belew et al. (2018)

## Discussion

In those studies, the overall prevalence of substandard/falsified medicines ranged from 0.5% to 88.4%, with an average of 22.6% (1718/7592) and of unregistered medicines 34.6% (108/312) (Tables 2–5). The lowest prevalence was reported in Gabon (0.5%)(Visser et al., 2015), whereas the highest prevalence was reported in Malawi (88.4%) (Chikowe et al., 2015), followed by Ghana and Togo (75%) (Osei-Safo et al., 2014). This study found a lower average prevalence of substandard/falsified medicines than the WHO report in Africa between 2013 and 2017 (42%) (WHO, 2017a). However, prevalence has increased from the 2018 report in African regions (18.7%) (Ozawa et al., 2018). Among the most commonly reported substandard, falsified, and unregistered medicines are antibiotics, antimalarial, anthelmintic, and antiprotozoal medicine. Antihypertensive medicines are also exposed to this problem (Chikowe et al., 2015; Hambisa et al., 2019; Kitutu, 2015; Koech et al., 2020; Njeri et al., 2021; Oli et al., 2020; Osei-Safo et al., 2014; Ozawa et al., 2018; Patricia et al., 2019; Visser et al., 2015).

The prevalence of antibiotics reported by those studies varied from 8.2% to 75%, with an average of 44.3% (712/1596), which were the most frequently found to be substandard/falsified medicines in Africa (Table 2). It is more than three times more prevalent than the 2018 reports in Low and Middle-Income Countries (12.4%) (Ozawa et al., 2018). In this study, the highest prevalence was reported in Nigeria (75%) (Oli et al., 2020), whereas the lowest prevalence was reported in Rwanda (8.2%) (Bizimana et al., 2022). Amoxicillin is the most substandard/falsified antibiotic. Other antibiotics like ciprofloxacin and co-trimoxazole have been exposed to substandard/falsified conditions in Kenya, Nigeria, Rwanda, and Congo (Hambisa et al., 2019; Kitutu, 2015; Koech et al., 2020; Njeri et al., 2021; Oli et al., 2020; Ozawa et al., 2018; Patricia et al., 2019).

Anti-malarial medicines, with an average prevalence of 15.6% (530/3530), are the second most commonly targeted medicines due to the high prevalence of malaria in African countries (Table 3). In this study, the highest prevalence of substandard, falsified, and unregistered anti-malarial medicines was reported in Malawi (88.4%) (Chikowe et al., 2015), whereas the lowest prevalence was reported in Gabon (0.5%) (Visser et al., 2015). However, the overall prevalence is lower than that reported in the 2018 in Low- and Middle-Income Countries (19.1%)(Ozawa et al., 2018). The most common anti-malarial medicines highly susceptible to falsification were artesunate, ACTs, and monotherapies based on artemisinin. However, studies conducted in Ghana in 2014 and 2016 revealed that the prevalence of substandard/falsified anti-malarial medicines decreased from 75% to 35% (Osei-Safo et al., 2014; Tivura et al., 2016).

Substandardisation/falsification also targets antihelminthic, antiprotozoal and antihypertensive medicines in African countries. The prevalence of substandard/falsified antihelmintic medicines was approximately 60.7% (91/150) (Table 5). The lowest prevalence of antihelminthic and antiprotozoal medicines was reported in Congo (26%) (Mwamba et al., 2016), whereas the highest prevalence was reported in Ethiopia (74.5%) (Suleman et al., 2014). Studies conducted in Ethiopia in 2014 and 2018 revealed that the prevalence of substandard/falsified antihelminthic and antiprotozoal medicines decreased from 74.5% to 33.33% (Belew et al., 2018; Suleman et al., 2014). Studies conducted in 10 African countries (Benin, Burkina Faso, Congo-Brazzaville, DRC, Niger, Senegal, Togo, Côte D’Ivoire, Guinea, and Mauritania) showed that the prevalence of substandard/falsified antihypertensive medicines accounted for approximately 16.3% (249/ 1530) (Faso, 2017).

The authors found that many factors make medicines a viable and attractive target for these problems, particularly in developing countries. Of the 312 medicine samples collected in Ghana, Togo, and Malawi, 34.6% (108/312) were not registered (Chikowe et al., 2015; Frimpong et al., 2018; Osei-Safo et al., 2014). Studies conducted in Ghana and Togo on 1132 samples (ACT formulation and Artemisinin-based monotherapy formulations) revealed that 75% of the samples had poor quality. Of these, non-registered medicines (84.7%) were more highly substandard/falsified than registered medicines (70.8%) (Osei-Safo et al., 2014). Compared with registered medicines (6.6%), nonregistered medicines had a higher failure rate (48.2%). Hence, non-registered medicines are more likely to be substandard/falsified. This is likely because higher registered medicines prices attract falsifiers. Weak regulatory systems have contributed to the distribution of substandard, falsified, and unregistered medicines in African countries (Chikowe et al., 2015; Frimpong et al., 2018; Osei-Safo et al., 2014).

According to studies conducted in Ghana and Togo, foreign manufacturing sources (77.5%) were more substandard/falsified than domestic manufacturing sources (77.3%) (Osei-Safo et al., 2014). This finding is supported by additional studies conducted in Ghana, which found that 59.4% of imported medicines failed (Frimpong et al., 2018). In the Democratic Republic of the Congo, of the 26% of substandard medicines, 78% were not authorised for marketing (Mwamba et al., 2016). The proportion of non-compliance was higher among medicines not permitted to be marketed and imported medicines. Additionally, the number of falsifiers is rising annually as a result of high-profit margins for falsified (90%) and substandard (65%) medicines compared with low-profit margins for genuine (45%) medicines (Frimpong et al., 2018; Khras, 2016; Mwamba et al., 2016; Osei-Safo et al., 2014).

Africa suffers from a lower standard of public health and socioeconomic effects due to the higher prevalence of substandard, falsified, and unregistered antibiotics, antimalarial, antihelminthic, and antiprotozoal medications, and an increase in the use of falsifiers. The quality and effectiveness of these medications were also extremely low. In addition to having a significant negative social and economic impact on patients and the healthcare system, the use of substandard, falsified, unlicensed, and unregistered medicines has numerous negative effects on public health, including increased morbidity, treatment failure, prolonged courses of therapy, death, and resistance to treatment (Akinyandenu, 2013; Beargie et al., 2019; Chikowe et al., 2015; Frimpong et al., 2018; Lalani et al., 2017; Mwamba et al., 2016; Osei-Safo et al., 2014; Peter et al., 2016; Tivura et al., 2016; WHO, 2017c)

The WHO*,* Osei-safo D.*,* et al., Frimpong G., et al., Peter A., et al., Khras KS., et al., and others found that weak or absence of drug regulatory authority and lack of regulation by exporters within free trade zones, high profit from falsified and substandard medicines, small pharmaceutical industries, high demand for curative and preventive medicines, high prices, inefficient or weak cooperation among stakeholders at national, regional, and global levels, lack of GMP in local pharmaceutical industries, and the knowledge, attitude, and practice of the health professional as well as the community toward substandard, falsified, unlicensed, and unregistered medicines leads to a higher prevalence in African countries than other countries (Akinyandenu*,*2013; Chikowe et al., 2015; Frimpong et al., 2018; Khras, 2016; Mwamba et al., 2016; Osei-Safo et al., 2014; Peter et al., 2016; WHO, 2017d).

## Limitations

Many studies lack a clear distinction between the prevalence of antibiotics and antimalarial medicines when samples of both medicines are collected and between substandard, falsified, unlicensed, and unregistered medicines. Some studies also failed to use random sampling design, and there are not nearly enough recent studies in Africa.

## Conclusion

In African countries, substandard, falsified, and unregistered medicines are very common; however, this issue is not being addressed. The most commonly reported substandard, falsified, and unregistered medicines in Africa are antibiotics, antimalarials, anthelmintics, and antiprotozoal drugs. A consistent supply of high-quality products, application of GMP to pharmaceutical industries, enhancement of registration, market legal permission, and importation standards, and effective enforcement of the existing drug laws in Africa are critical to controlling substandard, falsified, unlicensed, and unregistered medicines. In addition, professional educational development and awareness raising in communities are important to counter the problem. Preventing these problems is the primary duty of every responsible nation to save lives. Further research is required to assess the quality of drugs in Africa to inform policies to combat this serious health threat.

## Data Availability

All datasets used and analysed during the current study are available in the article.
